# Patient adherence, satisfaction and changes in anthropometric parameters with e-health versus in-person monitoring in metabolic bariatric surgery patients: A study protocol for a systematic review and non-inferiority meta-analysis of cohort studies

**DOI:** 10.1371/journal.pone.0313434

**Published:** 2025-01-24

**Authors:** Maíra Ribas Goulart, Karine Elisa Schwarzer Schmidt, Gustavo Waclawovsky, Izabele Vian

**Affiliations:** 1 Instituto de Cardiologia do Rio Grande do Sul/Fundação Universitária de Cardiologia (IC/FUC), Serviço de Nutrição e Dietética, Porto Alegre, Rio Grande do Sul, Brazil; 2 Laboratório de Investigação Clínica (LIC), Instituto de Cardiologia do Rio Grande do Sul/Fundação Universitária de Cardiologia (IC/FUC), Porto Alegre, Rio Grande do Sul, Brazil; Shiraz University of Medical Sciences, ISLAMIC REPUBLIC OF IRAN

## Abstract

**Background:**

Obesity is a risk factor for cardiovascular diseases and associated with reduced life expectancy metabolic bariatric surgery (MBS) is the treatment indicated when patients are unable to lose weight through lifestyle changes and medication alone. However, more evidence is necessary to show non-inferiority of e-health compared to in-person monitoring with regard to important parameters for the success of surgical treatment of obesity such as anthropometric changes.

**Methods and analyses:**

This review study will include cohort studies involving individuals with obesity and e-health or in-person patient monitoring before and after MBS. This study protocol was registered in the PROSPERO (CRD42023491051). We will conduct searches in the following databases: PubMed, EMBASE (Elsevier), Cochrane (CENTRAL), Web of Science, SCOPUS and CINAHL (EBSCO) and LILACS-VHL. We will also search databases in the gray literature. The primary outcomes will be changes in body mass index (BMI), body weight (kg) and body fat percentage (BF%) and patient adherence and satisfaction. The risk of bias of individual eligible studies will be assessed using the Newcastle-Ottawa Scale and the overall quality will be assessed using the GRADE tool. Our analyses will involve comparisons of mean differences or standardized mean differences across the groups using random-effects models and 95% confidence intervals. Statistical analyses will be performed with RStudio for Windows (v1.3.959) using R package meta (v3.6.1).

**Discussion and conclusion:**

Our study can offer evidence that shows the benefits of e-health patient monitoring of individuals undergoing MBS and supports scaling up this care modality to reduce waiting times and health care costs.

## Introduction

It is estimated that, by 2030, over 1 billion people will be living with obesity globally [1]. Obesity is associated with higher mortality, cardiovascular diseases, and reduced life expectancy [2,3]. The causes of obesity involve genetic, lifestyle, environmental and psychological factors [4].

Weight loss is essential to improve quality of life and decrease the risk of obesity-related comorbidities [5]. We can consider a feasible weight loss goal of 5–10% of the initial weight [6]. To achieve this goal, the patient must adhere to dietary and behavioral changes and, in many cases, pharmacological therapy [7,8]. Adhering to eating patterns that provide a calorie deficit, combined with the consumption of vegetables, fruits and whole grains, helps maintain weight loss [9,10]. When the patient does not reach the goal, even after adhering to treatment, metabolic bariatric surgery (MBS) may be indicated [11].

MBS is indicated for patients with a body mass index (BMI) ≥40 kg/m^2^, without associated comorbidities, or patients with a BMI ≥35 kg/m^2^, with associated comorbidities and MBS is a safe procedure [12]. MBS can bring other benefits such as diabetes remission, being more effective than drug therapy [13]; improve lipid profile and lower blood pressure [14]. The target weight loss after MBS, with no time limit, varies according to the surgical technique used. For Gastric bypass (Roux-en-Y) it is 30 to 35% and for Sleeve gastrectomy it is 25 to 30% [11], are the most used techniques in the world [15].

Multidisciplinary monitoring is essential for the long-term success of surgery [10,16,17]. This provides the team with greater knowledge about patients’ eating habits and nutritional needs. Based on this, dietary treatment can be effectively directed to ensure adequate nutritional intake and weight loss. Investigation into the presence of comorbidities and health conditions can help the team develop a therapeutic plan for patients, with the aim of minimizing complications and postoperative surgical infections and generating greater knowledge and self-care. Therefore, multidisciplinary monitoring can increase the chances of the patient achieving the weight loss goal and staying healthy, both pre- and postoperatively [10,18,19].

Monitoring via e-health by the multidisciplinary team is seen positively and is accepted by bariatric patients [20]. When a virtual access platform with educational information for the treatment of MBS is made available, patients show interest in accessing the content [21]. The carrying out consultations via video call or telephone call, using applications, sending text messages via WhatsApp, SMS or e-mail, can be alternatives to improve monitoring of metabolic bariatric surgery patients, generate greater knowledge about surgery and the necessary care and can be a tool for achieving behavioral changes [22,23].

Systematic reviews of cohort studies have weaker evidence than systematic reviews of randomized controlled trials (RCTs). However, in cases where patients need more time to show “real-life” results, without being in the controlled environment of RCTs, systematic reviews of longitudinal studies may be indicated [24]. Therefore, the aim of this systematic review and meta-analysis of cohort studies will be to assess the non-inferiority of e-health compared to in-person monitoring of individuals with obesity followed before or after bariatric surgery.

## Materials and methods

### Registry

This study will follow the PRISMA flow diagram [25] (S1 Appendix) and the Cochrane Handbook for Systematic Reviews of Interventions recommendations [26]. This study protocol was registered in the International Prospective Register of Systematic Reviews (PROSPERO) (www.crd.york.ac.uk/PROSPERO/, CRD42023491051). All databases used for the analyses of the proposed study outcomes will be made available on Mendeley Data repository as open access (https://data.mendeley.com/).

### Search strategy

We conducted a preliminary search in the PubMed database to test our research question using the FINER (Feasible, Interesting, Novel, Ethical, and Relevant) method and in the PROSPERO registry to search for ongoing systematic reviews with a similar focus before formulating a search strategy.

Two independent reviewers will conduct all searches for cohort studies in the databases PubMed, EMBASE (Elsevier), Cochrane (CENTRAL), Web of Science, SCOPUS and CINAHL (EBSCO). To expand the scale of this review, our searches will also include Latin American databases such as LILACS-VHL. To minimize any publication bias, we will also conduct searches in the gray literature, including OpenGrey, the WHO International Clinical Trials Registry Platform as well as preprint databases (preprints.org/, biorxiv.org and medrxiv.org). There will be no language or date restrictions. We will carefully review all articles retrieved before data collection, and authors will be contacted by email to obtain additional information as needed. In addition, we will cross-check references and perform Google Scholar searches. A manual review of all references of the included articles will be carried out. New searches will be conducted every six months to ensure retrieval of all eligible studies.

We predefined the terms and their entry terms for our searches from the DeCS/MeSH descriptors (PubMed, Cochrane and Embase). The key terms will include ‘bariatric surgery’ and ‘e-health’ or ‘telemedicine.’ We will perform searches for eligible studies using Boolean operators (OR, AND) when appropriate and allowed in each database (S2 Chart).

For the selection of eligible studies, all articles retrieved will be saved as ‘ris’, imported into Rayyan platform [27] and arranged in folders (by database, inclusion criteria and exclusion criteria). Two independent reviewers (KESS and MRG) will screen all studies based on their titles and abstracts.

Our review will include prospective and retrospective cohort studies involving adult candidates for metabolic bariatric surgery based on the American Society for Metabolic and Metabolic bariatric surgery (ASMBS) and International Federation for the Surgery of Obesity and Metabolic Disorders (IFSO) criteria [28] being monitored before and/or after metabolic bariatric surgery using e-health and in-person strategies.

ASMBS and IFSO [28] criteria for metabolic bariatric surgery:

- BMI 30–34.9 kg/m^2^ with type 2 diabetes mellitus and inadequate glycemic control despite optimal lifestyle and medical treatment;
- BMI ≥40 kg/m^2^ without obesity-related comorbidities;
- BMI ≥35 kg/m^2^ with obesity-related comorbidities.

The exclusion criteria will include ineligible population: children, adolescents, elderly (≥ 60 years old), animals, individuals not eligible for metabolic bariatric surgery or presenting other conditions such as cancer, pregnancy and problems that make self-care difficult; other study designs: cross-sectional, case-control, randomized clinical trials, study protocols, letter to the editor and reviews; other types of intervention: assessment or standardization of surgical techniques, *in vitro* studies; and other outcomes: aesthetic and other outcomes beyond those proposed for this review. Any disagreements will be resolved by a third reviewer (GW). Duplicate articles from searches will be excluded using Rayyan software during the selection of studies.

### Selection criteria

This systematic review and meta-analysis will include cohort studies evaluating e-health vs. in-person monitoring assessed by patient adherence and satisfaction and changes in anthropometric parameters in individuals with obesity undergoing metabolic bariatric surgery. We used PICOS strategy to develop the study design (Chart 1):

**Chart 1:** PICOS strategy of the systematic review.

Population	Adult (≥18 years of age) with obesity (Body Mass Index ≥30 kg/m^2^) undergoing metabolic bariatric surgery (laparoscopic sleeve gastrectomy (Sleeve) or laparoscopic gastric bypass).
Intervention	Monitoring via e-Health pre- or postoperatively applications, calls, video calls, tablet, computer, consultations or online classes.
Comparison	Standard and in-person multidisciplinary follow-up (clinic, outpatient, home).
Outcome	Primary: body weight, BMI, relative body adiposity, adherence and satisfaction with monitoring. Secondary: HbA1c, triglycerides, total cholesterol, HDL cholesterol and LDL cholesterol. Surgical approval and conversion, surgical delay, hospital readmission and Emergency Room visits.
Study	Prospective and retrospective cohort studies.

Overall, e-health monitoring involves practices that employ technologies to provide information, recommendations and remote care services to patients before and after metabolic bariatric surgery. Such technologies can include smartphone, computer and tablet applications, text messaging or video messaging via WhatsApp or SMS, e-mails, video calls, phone calls and pre-recorded sessions/recommendations. A single term—e-health—will be used to describe telemedicine, telehealth and mobile health (mhealth) [29,30].

### Data extraction and management

In preparation for data extraction, two reviewers (KESS and MRG) will carefully read the full text of all eligible studies to verify that they met the inclusion criteria. If a study is eligible for inclusion in the review, each reviewer will manually extract and compile the main data in a pre-structured database in Excel 2019 for Windows. For the extraction of data from studies with the outcomes of interest presented in graphs, we will use WebPlotDigitizer 4.6 to extract the data. Any disagreements between reviewers will be discussed and resolved by consensus.

The data extracted will be categorized into five main groups: study identification (authors and year of publication); participant characteristics (age, gender, type of surgery, lost to follow-up); materials and methods (sample size, follow-up duration, preoperative or postoperative monitoring, technology modality used); type of intervention and outcomes.

### Outcomes

The primary outcome of this review will be the rate of patient adherence and level of patient satisfaction with monitoring. Adhesion they will be defined as a percentage of presence in relation to the proposed segment time described by each author. Satisfaction concerns the extent to which the patient’s health expectations were met or not. It will be evaluated through the percentage of responses in relation to the questions present in the instruments proposed by each study. Changes in anthropometric parameters, including body weight (kg), total weight loss (TWL), excess weight loss (%EWL), BMI (kg/m^2^), excess of BMI loss (%EBMIL), and body fat percentage (BF%) will be evaluated.

In addition, we will evaluate secondary outcomes, i.e., changes in biochemical parameters known as traditional risk factors for cardiovascular diseases (glycated hemoglobin (HbA1c); triglycerides; total cholesterol; HDL cholesterol; and LDL cholesterol) as well as hospital-related and surgical outcomes (including surgery approval and conversion rate, surgery delays, hospital readmission rates and emergency room visits).

### Quality assessment

The quality of individual studies will be assessed using the Newcastle-Ottawa Quality Assessment Scale (NOS) instrument for observational studies [31]. This is a widely used tool for assessing quality of cohort studies and assigns points in the domains of ‘selection’ (4 points), ‘comparability’ (1 point) and ‘outcomes’ (3 points). The instrument is scored by awarding a point for each answer that is marked with an asterisk (*) for each item in the ‘selection’ and ‘outcomes’ domains. For the ‘comparability’ domain, a maximum of two asterisks (**) can be marked. The total score ranges from no asterisk (poor quality) to nine asterisks (good quality).

The strength of the body of evidence of this review will be assessed using the GRADE (Grading of Recommendations, Assessment, Development and Evaluation) tool [32,33]. This tool classifies the quality of evidence into four levels (high, moderate, low and very low) based on the assessment of confidence in specific estimates in five domains: methodological limitations (risk of bias); inconsistency; indirectness of evidence; imprecision; and publication bias. However, based on the GRADE assessment system, the evidence from reviews of observational studies (as in our study) is primarily rated as ‘low quality.’ If such studies yield large effects (and these effects cannot be attributed to potential confounders) and/or all plausible biases from observational may be working to underestimate an apparent benefit (confounders and other biases act towards reducing the effect estimate), then we will rate the evidence as ‘moderate quality’ [33].

### Data synthesis

Statistical analyses will be performed to test that e-health monitoring is not inferior to in-person monitoring in metabolic bariatric surgery patients assessed by patient adherence and satisfaction and changes in body composition and anthropometric parameters. When the outcomes of studies are measured in the same unit, a summary effect estimate will be presented as mean difference (MD) and 95% confidence interval (95% CI). Categorical variables data will be presented as relative risk (RR) and (95% CI). When the outcomes are in different units, if appropriate, they will be converted into a standard unit and a summary effect estimate will be presented as standardized mean difference (SMD) and 95% CI taking into account the effect size of the intervention in relation to the variability in individual studies. As SMD offers a ‘generic interpretation’ (absolute unit, SMD), will be examined SMD can be converted into the unit of the outcome measure most familiar or into proportion (%) to present data in a usual parameters used.

To summarize the data, given potential heterogeneity across studies (including differences in population characteristics or methods), random-effects model and inverse variance method will be used to pool together MD or SMD of individual studies. Since the 95% CI from random effects refer to uncertainty in the location of the mean of effects in the different studies, values will be calculated for a 95% prediction interval (95% PI) (S3 Chart) as they reflect the interval of uncertainty of the effects to be expected in future studies [34]. To assess consistency across studies, the degree of heterogeneity (relative variability in effect estimates attributed to heterogeneity) will be tested with the Higgins inconsistency test (I2) for every pairwise comparison [35,36] (S4 Chart). To explore the heterogeneity (p<0.05), will be analyzed subgroup analyses or meta-regression (≥10 studies) for effect modifiers with normal distribution in a quartile-quartile plot (qq-plot) and confirm it with the Shapiro-Wilk test (p>0.05) [37]. In addition, to remove discrepant data from the meta-analysis, forest plots will be constructed to visualize the effect estimate of individual studies and detect outliers based on non-CI overlapping that is due to heterogeneity [36]. Potential effect modifiers, including age, BMI, gender, monitoring duration, rate of patient adherence during the proposed duration of monitoring and geographic locations will be analyzed separately. If there is significant heterogeneity between the studies that cannot be explained, will not be realized a meta-analysis and estimates of intervention effects from the studies will be presented individually. Considering that selective publication and/or suppression of publication of certain results may cause bias and consequently reduce the validity of results [38], if applicable (≥10 studies; ≥1 study with statistically significant data; studies with different sample sizes), will be perform the Egger’s test using a funnel plot to assess potential publication bias [39,40]. If publication bias is detected (Egger’s test, p<0.1), the trim and fill method will be conducted to identify and correct the funnel plot’s asymmetry and the adjusted data will be added to the description of the original data (after trimmed and imputed data by the trim and fill method) [41,42]. Alternative analyses to the primary analysis (sensitivity analysis) will be carried out to determine the robustness of our decisions, including missing value imputation method used, inclusion of studies with high risk of bias, data from conference abstracts and others [43]. All measures of dispersion presented as CI or standard errors (SE) will be converted into standard deviations (SD) (SD = SE * √n) before the analysis. For eligible studies that do not have SD for the differences, SD will be estimated using an imputed correlation coefficient (CC) of 0.5 [44]. We will use the following CC equation (section 6.5.2.8, Cochrane Handbook): Δ SD = √ SD2 baseline + SD2 final–(2 * CC * SD baseline * SD final).

Will be used a margin of ±10% (S1 Table) with no time frame to assess non-inferiority of e-health monitoring compared to in-person monitoring. Secondary outcomes will be also evaluated (S1 Chart). All statistical tests will be two-tailed with a significance level of 0.05. All dispersion measures presented as SEs will be converted into SDs using Microsoft Excel (2019) before the analyses. Data modelizations will be performed with RStudio for Windows (v1.3.959) using R package meta (v3.6.1) [45].

## Discussion

The World Health Organization states that digital technologies are an integral part of everyday life and there is a huge scope for the use of digital health solutions [46]. Technology as a tool to assist in health treatment can bring countless benefits, from providing savings for patients due to reduced transportation costs and absences from work to facilitating communication between patients and professionals [23,47]. The role of e-health within health systems should be seen as part of technological evolution and in a broad context of access to health; it should encompass clinical decision support, health information sharing, health education and assistance between patients and professionals [48].

Advances in technology as a health tool can help bariatric patients to better adhere to treatment and avoid weight regain [49,50]. According to a systematic review, 1 in 6 patients experience weight regain of more than 10% [51], and postoperative monitoring is essential to minimize this risk. The recommendations of the multidisciplinary team to follow the nutritional plan and maintain adequate intake of supplements, regular physical activity of 150 to 300 minutes per week, and psychological support enhance the results of surgery and keep the patient healthy [10,19].

We cite as a limitation of this study the evidence of lower strength when compared to systematic reviews conducted with RCTs, and also to possible confounding factors inherent in observational studies, such as confounding bias. However, a review published by Coldebella et al. [22] evaluated the use of telemedicine in the provision of health services for bariatric patients through longitudinal studies and RCTs and did not obtain definitive conclusions about the outcomes studied. RCTs evaluating the use of e-health in bariatric patients have a short follow-up period [49,52,53], we know that the treatment of bariatric patients is long, involves monitoring both pre- and post-operatively, and avoiding weight regain over the months is one of the main challenges faced by patients.

Therefore, a systematic review with meta-analysis of cohort studies will be carried out, as we believe that bariatric patients require long-term monitoring and that the results obtained without the controlled RCT environment may fill possible gaps in the literature on the subject, especially regarding the use of technologies as a tool for long-term monitoring of bariatric patients.

## Conclusions

Demonstrating the benefits of e-Health in the surgical treatment of obesity as an alternative to in-person monitoring will enable broad access to surgery without compromising monitoring by the multidisciplinary team, since regardless of the monitoring model offered, patients will have the opportunity to achieve the results proposed by the treatment, remain healthy and have a better quality of life.

## Supporting information

S1 Table Values used to determine ±10% as the cutoff point to describe the non-inferiority between e-Health monitoring versus in-person monitoring of our systematic review. (DOCX)

S1 Chart Definition for the outcomes proposed in our systematic review. (DOCX)

S2 Chart Search strategy via databases and registries. (DOCX)

S3 Chart Formula for the prediction interval. (DOCX)

S4 Chart Formula and interpretation of heterogeneity in meta-analysis. (DOCX)

S1 Appendix Figure study flowchart. (DOCX)

S2 Appendix PRISMA-P 2015 checklist. (DOCX)
