# Supplementary material for: Patient adherence, satisfaction and changes in anthropometric parameters with e-health versus in-person monitoring in metabolic bariatric surgery patients: A study protocol for a systematic review and non-inferiority meta-analysis of cohort studies
Source: PLoS One. 2025 Jan 24;20(1):e0313434. doi: 10.1371/journal.pone.0313434 (PMC11761637; doi:10.1371/journal.pone.0313434)
Supplement: S1 Table — (DOCX) [file pone.0313434.s001.docx]

**S1 Table.** Values used to determine ±10% as the cutoff point to describe the non-inferiority between e-Health monitoring versus in-person monitoring of our systematic review.

| **Variables** |  | |
| --- | --- | --- |
|  |  |  |
| **Reduction in BMI (points)** | **Bypass (Roux-em-Y)** | **Sleeve Gastrectomy** |
| < 1 year [1] | -9,0 | -8,3 |
| 1 to 2 years [2] | -15,8 | -12,0 |
| > 2 years [2] | - 16,1 | - 11,3 |
| **Reduction in total body mass (%)** | **Bypass (Roux-em-Y)** | **Sleeve Gastrectomy** |
| < 1 year [3] | 26,0 | 18,5 |
| 1 to 2 years [4] | 32,6 | 27,1 |
| > 2 years [5] | 32,5 | 25,6 |
| **Decrease in Adiposity (%)** | **Bypass (Roux-em-Y)** | **Sleeve Gastrectomy** |
| < 1 year [2] | 9,26 | 8,0 |
| 1 to 2 years [2] | 14,4 | 12,9 |
| > 2 years [6, 7] | 23,1 | 16,7 |

REFERENCES

1. Le Foll D, Lechaux D, Rascle O, Cabagno G. Weight loss and quality of life after bariatric surgery: a 2-year longitudinal study. Surg Obes Relat Dis. 2020;16(1):56-64. doi: [10.1016/j.soard.2019.10.010](https://pubmed.ncbi.nlm.nih.gov/31753793/) PMID: [31753793](https://pubmed.ncbi.nlm.nih.gov/31753793/)

2. Haghighat N, Ashtari-Larky D, Aghakhani L, Asbaghi O, Hoseinpour H, Hosseini B, et al. How Does Fat Mass Change in the First Year After Bariatric Surgery? A Systemic Review and Meta-Analysis. Obes Surg. 2021;31(8):3799-821. doi: [10.1007/s11695-021-05512-9](https://doi.org/10.1007/s11695-021-05512-9) PMID: [34089442](https://pubmed.ncbi.nlm.nih.gov/34089442/)

3. Lee JH, Nguyen QN, Le QA. Comparative effectiveness of 3 bariatric surgery procedures: Roux-en-Y gastric bypass, laparoscopic adjustable gastric band, and sleeve gastrectomy. Surg Obes Relat Dis. 2016;12(5):997-1002. doi: [10.1016/j.soard.2016.01.020](https://doi.org/10.1016/j.soard.2016.01.020) PMID: [27220823](https://pubmed.ncbi.nlm.nih.gov/27220823/)

4. Nicoletti CF, Camelo JS, Jr., dos Santos JE, Marchini JS, Salgado W, Jr., Nonino CB. Bioelectrical impedance vector analysis in obese women before and after bariatric surgery: changes in body composition. Nutrition. 2014;30(5):569-74.  doi: [10.1016/j.nut.2013.10.013](https://doi.org/10.1016/j.nut.2013.10.013) PMID:[24698348](https://pubmed.ncbi.nlm.nih.gov/24698348/)

5. Enochs P, Bull J, Surve A, Cottam D, Bovard S, Bruce J, et al. Comparative analysis of the single-anastomosis duodenal-ileal bypass with sleeve gastrectomy (SADI-S) to established bariatric procedures: an assessment of 2-year postoperative data illustrating weight loss, type 2 diabetes, and nutritional status in a single US center. Surg Obes Relat Dis. 2020;16(1):24-33. doi: [10.1016/j.soard.2019.10.008](https://doi.org/10.1016/j.soard.2019.10.008) PMID: [31753795](https://pubmed.ncbi.nlm.nih.gov/31753795/)

6. Maïmoun L, Aouinti S, Puech M, Lefebvre P, Deloze M, de Santa Barbara P, et al. Changes in Lean Tissue Mass, Fat Mass, Biological Parameters and Resting Energy Expenditure over 24 Months Following Sleeve Gastrectomy. Nutrients. 2023;15(5). doi: [10.3390/nu15051201](https://doi.org/10.3390/nu15051201) PMID: [36904198](https://pubmed.ncbi.nlm.nih.gov/36904198/)

7. Walker E, Elman M, Takemoto EE, Fennern E, Mitchell JE, Pories WJ, et al. Bariatric Surgery Among Medicare Subgroups: Short- and Long-Term Outcomes. Obesity (Silver Spring). 2019;27(11):1820-7. doi: [10.1002/oby.22613](https://doi.org/10.1002/oby.22613) PMID: [31562705](https://pubmed.ncbi.nlm.nih.gov/31562705/).
