# Supplementary material for: Patient adherence, satisfaction and changes in anthropometric parameters with e-health versus in-person monitoring in metabolic bariatric surgery patients: A study protocol for a systematic review and non-inferiority meta-analysis of cohort studies
Source: PLoS One. 2025 Jan 24;20(1):e0313434. doi: 10.1371/journal.pone.0313434 (PMC11761637; doi:10.1371/journal.pone.0313434)
Supplement: S1 Chart — (DOCX) [file pone.0313434.s002.docx]

| **S1 Chart.**  Definition for the outcomes proposed in our systematic review. | |
| --- | --- |
| Adhesion  (e-Health / In-person) | They will be defined as a percentage of presence in relation to the proposed segment time described by each author. |
| Satisfaction with monitoring  (e-Health / In-person) | Satisfaction concerns the extent to which the patient's health expectations were met or not. Two people who receive the same care, but who have different expectations about how that care should be provided, may give different satisfaction ratings due to their different expectations. [8]. |
| Total body mass | Total body mass is made up of clusters of bones, muscles, fats and other tissues [9]. And regarding the anatomical aspect, it is made up of: muscle mass, fat mass, bone mass and residual mass [10]. |
| BMI | The Body Mass Index (BMI) is calculated from weight (kg) divided by height² (meters) [11]. |
| Body adiposity | Amount of total body adipose tissue. The percentage of total body fat will be classified as abnormal when the value will be ≥ 20% (men) and ≥ 32 (women) between 20 and 39 years old and ≥ 22% (men) and ≥ 34 (women) between 40 and 59 years [12]. |
| Laboratory tests | Values in mg/dL or mmol/L will be considered, the interpretation will be in accordance with the cutoff points referenced in the studies included in the analysis, if this is not possible or there is a discrepancy between the cutoff points, the values will be interpreted in accordance with the recommendations for normality recommended by the American Heart Association and the American Diabetes Association. [13, 14] |
| Hospital outcomes (surgical approval and conversion, surgical delay, hospital readmission and Emergency Room visits). | They will be defined in percentage (surgical conversion, hospital readmission and Emergency Room visits) and minutes, hours or days (surgical delay). |

REFERENCES

8. Agency. for Healthcare Research and Quality. What is patient ex‐ perience? 2017. Nov [Cited 2023 november 29] <https://www.ahrq.gov/cahps/about-cahps/patient-experience/index.html>.

9. McARDLE, al e. Nutrição, controle de peso e exercício. 3 ed. Rio de Janeiro : Medsi, 1990.

10. Pollock M, Wilmore HJ. Exercícios na saúde e na doença: avaliação e prescrição para prevenção e reabilitação. 2. ed. Rio de Janeiro: MEDSI,1993.

11. Keys A, Fidanza F, Karvonen MJ, Kimura N, Taylor HL. Indices of relative weight and obesity*. International Journal of Epidemiology. 2014;43(3):655-65. doi: [10.1093/ije/dyu058](https://doi.org/10.1093/ije/dyu058) PMID: [24691951](https://pubmed.ncbi.nlm.nih.gov/24691951/)

12. Gallagher D, Heymsfield SB, Heo M, Jebb SA, Murgatroyd PR, Sakamoto Y. Healthy percentage body fat ranges: an approach for developing guidelines based on body mass index. Am J Clin Nutr. 2000;72(3):694-701. doi: [10.1093/ajcn/72.3.694](https://doi.org/10.1093/ajcn/72.3.694) PMID: [10966886](https://pubmed.ncbi.nlm.nih.gov/10966886/)

13. Grundy SM, Stone NJ, Bailey AL, Beam C, Birtcher KK, Blumenthal RS, et al. 2018 AHA/ACC/AACVPR/AAPA/ABC/ACPM/ADA/AGS/APhA/ASPC/NLA/PCNA Guideline on the Management of Blood Cholesterol: A Report of the American College of Cardiology/American Heart Association Task Force on Clinical Practice Guidelines. Circulation. 2019;139(25):e1082-e143. doi: [10.1016/j.jacc.2018.11.002](https://doi.org/10.1016/j.jacc.2018.11.002) PMID: [30423391](https://pubmed.ncbi.nlm.nih.gov/30423391/)

14. American Diabetes Association. 2. Classification and Diagnosis of Diabetes: *Standards of Medical Care in Diabetes-2020*. Diabetes Care. 2020 Jan;43(Suppl 1):S14-S31. doi: [10.2337/dc20-S002](https://pubmed.ncbi.nlm.nih.gov/31862745/). PMID: [31862745](https://pubmed.ncbi.nlm.nih.gov/31862745/).
