# Supplementary material for: Patient adherence, satisfaction and changes in anthropometric parameters with e-health versus in-person monitoring in metabolic bariatric surgery patients: A study protocol for a systematic review and non-inferiority meta-analysis of cohort studies
Source: PLoS One. 2025 Jan 24;20(1):e0313434. doi: 10.1371/journal.pone.0313434 (PMC11761637; doi:10.1371/journal.pone.0313434)
Supplement: S2 Chart — (DOCX) [file pone.0313434.s003.docx]

| **S2 Chart.** Search strategy via databases and registries. |
| --- |
| **MEDLINE (PubMed)** |
| **Telemedicine:** (Tele-Referral OR Tele Referral OR Tele-Referrals OR Virtual Medicine OR Medicine, Virtual OR telemedicine OR Tele-ICU OR Mobile Health OR Health, Mobile OR mHealth OR Telehealth OR eHealth OR Tele-Intensive Care OR Tele Intensive Care) AND  **Bariatric Surgery:** (Stomach Stapling OR Stapling, Stomach OR Bariatric Surgeries OR Surgical Procedures, Bariatric OR Surgical Procedure, Bariatric OR Procedures, Bariatric Surgical OR Surgeries, Metabolic OR Surgery, Metabolic OR Bariatric Surgical Procedures OR Surgery, Bariatric OR Bariatric Surgical Procedure OR Metabolic Surgery OR Metabolic Surgeries OR Surgeries, Bariatric OR Procedure, Bariatric Surgical) |
| **Cochrane** |
| **Telemedicine:** (Tele-Referral OR Tele Referral OR Tele-Referrals OR Virtual Medicine OR Medicine, Virtual OR telemedicine OR Tele-ICU OR Tele ICU OR Mobile Health OR Health, Mobile OR mHealth OR Telehealth OR eHealth OR Tele-Intensive Care OR Tele Intensive Care) AND  **Bariatric Surgery:** (Stomach Stapling OR Stapling, Stomach OR Bariatric Surgeries OR Surgical Procedures, Bariatric OR Surgical Procedure, Bariatric OR Procedures, Bariatric Surgical OR Surgeries, Metabolic OR Surgery, Metabolic OR Bariatric Surgical Procedures OR Surgery, Bariatric OR Bariatric Surgical Procedure OR Metabolic Surgery OR Metabolic Surgeries OR Surgeries, Bariatric OR Procedure, Bariatric Surgical) |
| **LILACS** |
| 1. **English**   **Telemedicine:** Telemedicine AND  **Bariatric Surgery:** Bariatric surgery   1. **Portuguese (Brazil)**   **Telemedicina:** (Telemedicina OR Ciber Saúde OR Cibersaúde OR eSaúde OR Medicina Virtual OR mSaúde OR Serviço de Telemedicina OR Serviço de Telessaúde OR Serviços de eSaúde OR Serviços de Telemedicina OR Serviços de Telessaúde OR Telessaúde) AND  **Cirurgia Bariátrica:** cirurgia bariátrica   1. **Espanish**   **Telemedicina: (**Telemedicina OR Agenda de eSalud OR Ciber Salud OR Componentes de eSalud OR eSalud OR mSalud OR Proyectos de Telemedicina OR Salud Digital OR Salud Mueble OR Servicio de Telemedicina OR Servicio de Telesalud OR Servicios de eSalud OR Servicios de Telemedicina OR Teleasistencia OR Telesalud OR Teleservicios de Salud OR uSalud AND  **Cirugía Bariátrica:** Cirugía Bariátrica |
| **EMBASE** |
| **Telemedicine:** (tele medicine OR virtual medicine OR telemedicine OR e-health OR Ehealth OR tele-health OR telehealth OR telecare) AND  **Bariatric Surgery: (**bariatric surgery OR bariatric operation OR bariatric operations OR bariatric procedure OR bariatric procedures OR bariatric surgical procedure OR bariatric surgical procedures OR metabolic surgery OR obesity operation OR obesity surgery OR obesity surgical treatment OR surgery, bariatric OR weight loss operation OR weight loss surgery OR weight reduction operation OR weight reduction surgery OR bariatric surgery) |
| **CINAHL (EBSCO)** |
| **Telemedicine:** (Telemedicine OR Telehealth OR Remote Consultation) AND  **Bariatric Surgery:** Bariatric Surgery OR Gastric Bypass OR Gastroplasty OR Jejunoileal Bypass. |
| **WEB OF SCIENCE** |
| **Telemedicine:** (Tele-Referral OR Tele Referral OR Tele-Referrals OR Virtual Medicine OR Medicine, Virtual OR telemedicine OR Tele-ICU OR Mobile Health OR Health, Mobile OR mHealth OR Telehealth OR eHealth OR Tele-Intensive Care OR Tele Intensive Care) AND  **Bariatric Surgery:** (Stomach Stapling OR Stapling, Stomach OR Bariatric Surgeries OR Surgical Procedures, Bariatric OR Surgical Procedure, Bariatric OR Procedures, Bariatric Surgical OR Surgeries, Metabolic OR Surgery, Metabolic OR Bariatric Surgical Procedures OR Surgery, Bariatric OR Bariatric Surgical Procedure OR Metabolic Surgery OR Metabolic Surgeries OR Surgeries, Bariatric OR Procedure, Bariatric Surgical) |
| **SCOPUS** |
| **Telemedicine:** (telemedicine OR Mobile Health) AND  **Bariatric Surgery:** (Bariatric Surgeries OR Surgeries, Metabolic OR Surgery, Bariatric OR Metabolic Surgery OR Metabolic Surgeries) |
| **GRAY LITERATURE** |
| **OpenGrey:** Bariatric Surgery AND Telemedicine  **WHO:** Bariatric Surgery OR Metabolic Surgery AND Telemedicine OR telehealth OR e-health  **preprints.org: Terms 1:** Bariatric Surgery AND Telemedicine  **biorxiv.org:** Bariatric Surgery AND Telemedicine  **medrxiv.org:** Bariatric Surgery AND Telemedicine |
