# Supplementary material for: Patient adherence, satisfaction and changes in anthropometric parameters with e-health versus in-person monitoring in metabolic bariatric surgery patients: A study protocol for a systematic review and non-inferiority meta-analysis of cohort studies
Source: PLoS One. 2025 Jan 24;20(1):e0313434. doi: 10.1371/journal.pone.0313434 (PMC11761637; doi:10.1371/journal.pone.0313434)
Supplement: S3 Chart — (DOCX) [file pone.0313434.s004.docx]

**S3 Chart.** Formula for the prediction interval.

| **Formula** |
| --- |
| 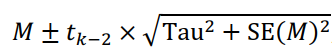 |
| Where M is the summary mean of a random-effects meta-analysis, t*_k_*_−2_ is the 95% percentile of a t distribution with k–2 degrees of freedom, *k* is the number of studies, *Tau^2^* is the estimated amount of heterogeneity and SE(M) is the standard error of the summary mean. |
|  |
