# Supplementary material for: Patient adherence, satisfaction and changes in anthropometric parameters with e-health versus in-person monitoring in metabolic bariatric surgery patients: A study protocol for a systematic review and non-inferiority meta-analysis of cohort studies
Source: PLoS One. 2025 Jan 24;20(1):e0313434. doi: 10.1371/journal.pone.0313434 (PMC11761637; doi:10.1371/journal.pone.0313434)
Supplement: S4 Chart — (DOCX) [file pone.0313434.s005.docx]

| **S4 Chart.** Formula and interpretation of heterogeneity in meta-analysis**.** | |
| --- | --- |
| **Interpretation** | ***I*^2^ formula** |
| - **0% to 40%:** might not be important | 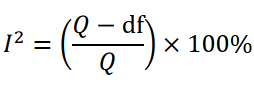 |
| - **30% to 60%:** may represent moderate heterogeneity* |  |
| - **50% to 90%:** may represent substantial heterogeneity* |  |
| - **75% to 100%:** considerable heterogeneity* |  |
| * The importance of the observed value of *I^2^* depends on the magnitude and direction of effects and strength of evidence for heterogeneity (confidence interval for *I^2^*: uncertainty of *I*^2^ is substantial when the number of studies is small). Q is the chi-squared statistic and *df* (degrees of freedom) is the degree of freedom. *I^2^* describes the percentage of variability in effect estimates that is due to heterogeneity. | |
|  | |
