# Supplementary material for: Patient adherence, satisfaction and changes in anthropometric parameters with e-health versus in-person monitoring in metabolic bariatric surgery patients: A study protocol for a systematic review and non-inferiority meta-analysis of cohort studies
Source: PLoS One. 2025 Jan 24;20(1):e0313434. doi: 10.1371/journal.pone.0313434 (PMC11761637; doi:10.1371/journal.pone.0313434)
Supplement: S1 Appendix — (DOCX) [file pone.0313434.s006.docx]

**S1 Appendix**

**Figure study flowchart**

**Identification of studies via databases and registers**

**Identification of studies via other methods**

Records identified from*:

Databases (n = )

Registers (n = )

Records identified from:

Websites (n = )

Organisations (n = )

Citation searching (n = )

etc.

Records removed *before screening*:

Duplicate records removed (n = )

Records marked as ineligible by automation tools (n = )

Records removed for other reasons (n = )

**Identification**

Records screened

(n = )

Records excluded**

(n = )

Reports not retrieved

(n = )

Reports sought for retrieval

(n = )

Reports not retrieved

(n = )

Reports sought for retrieval

(n = )

**Screening**

Reports excluded:

Reason 1 (n = )

Reason 2 (n = )

Reason 3 (n = )

etc.

Reports assessed for eligibility

(n = )

Reports excluded:

Reason 1 (n = )

Reason 2 (n = )

Reason 3 (n = )

etc.

Reports assessed for eligibility

(n = )

Studies included in review

(n = )

Reports of included studies

(n = )

**Included**

*Consider, if feasible to do so, reporting the number of records identified from each database or register searched (rather than the total number across all databases/registers).

**If automation tools were used, indicate how many records were excluded by a human and how many were excluded by automation tools.

*From:*  Page MJ, McKenzie JE, Bossuyt PM, Boutron I, Hoffmann TC, Mulrow CD, et al. The PRISMA 2020 statement: an updated guideline for reporting systematic reviews. BMJ 2021;372:n71. doi: 10.1136/bmj.n71. For more information, visit: <http://www.prisma-statement.org/>
